# Cytomegalovirus, midlife inflammation, and subclinical cardiovascular disease: the SWAN Study

**DOI:** 10.1093/geroni/igaf122.2961

**Published:** 2025-12-31

**Authors:** Aleda Leis, Michelle Hood, Wade Sanders, Laura McLees, Carrie Karvonen-Gutierrez

**Affiliations:** University of Michigan, Ann Arbor, Michigan, United States; University of Michigan School of Public Health, Ann Arbor, Michigan, United States; University of Michigan, Ann Arbor, Michigan, United States; University of Michigan, Ann Arbor, Michigan, United States; University of Michigan, Ann Arbor, Michigan, United States

## Abstract

Cytomegalovirus (CMV) is a common beta herpesvirus with over half of US adults infected by age 40. Following an often asymptomatic acute infection, the virus remains latent with the potential for reactivation across the lifecourse. We examined associations of CMV seropositivity with the inflammatory marker c-reactive protein and carotid intima media thickness (cIMT) in the Michigan site of the Study of Women’s Health Across the Nation (SWAN). Qualitative serum CMV immunoglobulin G (IgG) and M (IgM) assays were conducted in 100 pre- or peri-menopausal women (50 White, 50 Black, mean age 48.3 years) with carotid artery ultrasounds approximately 10 years later. Latent CMV seropositivity was defined as being IgG positive and IgM negative. Overall, 61% of samples were seropositive for latent CMV infection, with higher CMV seropositivity in Black (78%) than White (44%) women. Those with latent CMV seropositivity had almost two times higher C-reactive protein levels those without (8.7mg/L (standard deviation (SD):14.4) vs. 4.6mg/L (SD:4.7)). Latent CMV seropositivity was associated with future subclinical cardiovascular risk. Those with CMV seropositivity had larger (worse) average carotid intima media thickness (cIMT) (mean 0.86mm (SD:0.12) vs. 0.76mm (SD:0.10); p < 0.001) and maximal cIMT (mean 1.01mm (SD:0.14) vs. 0.90mm (SD:0.1); p < 0.001) compared to those who were not CMV seropositive. Results were similar when stratified by race. Together, the results suggest CMV seropositivity may be associated with greater inflammation and cardiovascular risk as women enter older adulthood.

